# First report on in-depth genome and comparative genome analysis of a metal-resistant bacterium *Acinetobacter pittii* S-30, isolated from environmental sample

**DOI:** 10.3389/fmicb.2024.1351161

**Published:** 2024-04-29

**Authors:** Rajnish Prakash Singh, Ayushi Sinha, Sushanta Deb, Kiran Kumari

**Affiliations:** ^1^Department of Biotechnology, Jaypee Institute of Information Technology, Noida, India; ^2^Department of Veterinary Microbiology and Pathology, Washington State University (WSU), Pullman, WA, United States; ^3^Department of Bioengineering and Biotechnology, Birla Institute of Technology, Mesra, Ranchi, Jharkhand, India

**Keywords:** genomics, PCR, virulence, pangenome, CAZymes

## Abstract

A newly isolated bacterium *Acinetobacter pittii* S-30 was recovered from waste-contaminated soil in Ranchi, India. The isolated bacterium belongs to the ESKAPE organisms which represent the major nosocomial pathogens that exhibit high antibiotic resistance. Furthermore, average nucleotide identity (ANI) analysis also showed its closest match (>95%) to other *A. pittii* genomes. The isolate showed metal-resistant behavior and was able to survive up to 5 mM of ZnSO_4_. Whole genome sequencing and annotations revealed the occurrence of various genes involved in stress protection, motility, and metabolism of aromatic compounds. Moreover, genome annotation identified the gene clusters involved in secondary metabolite production (biosynthetic gene clusters) such as arylpolyene, acinetobactin like NRP-metallophore, betalactone, and hserlactone-NRPS cluster. The metabolic potential of *A. pittii* S-30 based on cluster of orthologous, and Kyoto Encyclopedia of Genes and Genomes indicated a high number of genes related to stress protection, metal resistance, and multiple drug-efflux systems etc., which is relatively rare in *A. pittii* strains. Additionally, the presence of various carbohydrate-active enzymes such as glycoside hydrolases (GHs), glycosyltransferases (GTs), and other genes associated with lignocellulose breakdown suggests that strain S-30 has strong biomass degradation potential. Furthermore, an analysis of genetic diversity and recombination in *A. pittii* strains was performed to understand the population expansion hypothesis of *A. pittii* strains. To our knowledge, this is the first report demonstrating the detailed genomic characterization of a heavy metal-resistant bacterium belonging to *A. pittii.* Therefore, the *A. pittii* S-30 could be a good candidate for the promotion of plant growth and other biotechnological applications.

## Introduction

The genus *Acinetobacter* was first reported by Brisou and Prevot (1954) and members are known as high-priority nosocomial pathogens responsible for severe infections among immunocompromised patients (Howard et al., 2012). The infections caused by *Acinetobacter* spp. seem untreatable as these microorganisms exhibit high potential to develop resistance to a wide range of antibiotics (Wong et al., 2019). Among the *Acinetobacter* genus, *A. pittii* is a close relative of *Acinetobacter baumanii* which is increasingly recognized as a significant cause of hospital-acquired infections (HAI) (Chusri et al., 2014) and is an opportunistic pathogen capable of causing a potentially fatal infection (Wisplinghoff et al., 2012; Chusri et al., 2014). *Acinetobacter* spp. along with *Enterococcus faecium*, *Staphylococcus aureus*, *Klebsiella pneumoniae*, *Pseudomonas aeruginosa*, and *Enterobacter* spp. belongs to the ESKAPE pathogens. These ESKAPE pathogens are also found in beach sand and intertidal beach water, wastewater systems like industrial and municipal, raw and ready-to-eat foods, and soils of human dumpsites. It was reported that around 46.4% of ESKAPE bacteria are from hospitals, sewerage plants, and pharmaceutical factories, resistant to many antibiotics (Hrenovic et al., 2017; Amarasiri et al., 2020). ESKAPE pathogens develop resistant mechanism against many antibiotics such as lipopeptides, β-lactams, macrolides, oxazolidinones, fluoroquinolones, tetracyclines, and other antibiotic combinations are through genetic mutation as well as acquisition of mobile genetic elements (MGEs) (Beatson and Walker, 2014; Naylor et al., 2018). The most common AMR (antimicrobial resistance) mechanism is that ESKAPE pathogens produce enzymes that irreversibly destroy or neutralize antibiotics. These enzymes belong to Gram-negative pathogens and act by a mechanism like destroying active antibiotic sites and also modifying the key elements of antibiotics to inhibit bacterial target site interaction (De Oliveira et al., 2020).

A recent study demonstrated that *A. pittii* was the dominant species causing invasive *Acinetobacter* infection in Japan (Kiyasu et al., 2020); however, studies have shown that a few of the *Acinetobacter* species have also been recovered from various environmental sources such as activated sludge, sewage, dump sites, raw wastewater, and hydrocarbon-contaminated areas (Doughari et al., 2011). Moreover, the contamination of the environment with heavy metals and other pollutants poses a serious threat to human health and the environment. At low levels, heavy metals show high toxicity against most microorganisms (Lemire et al., 2013), however, microorganisms including bacteria have evolved several molecular mechanisms in order to cope with heavy metal toxicity (Lemire et al., 2013). *A. pittii* is a Biosafety Level (BSL) 2 pathogen capable of causing potentially fatal infection, most commonly in immunocompromised patients in clinical settings. The findings of *A. pittii* from the International Space Station (ISS) illustrate its potential for human infection in this extreme environment (Urbaniak et al., 2021). This indicates a propensity for rapid adaptation to conditions aboard the ISS and acquiring additional genes that could contribute to antibiotic resistance. Previous studies showed that MDR (multidrug resistance) *A. pittii* including those resistant to antibiotics such as quinolones, carbapenems, penicillins, and cephalosporins was reported across continents (Brasiliense et al., 2019; Cosgaya et al., 2019; Chopjitt et al., 2021). More than 50 *A. pittii* genome sequences are available on the National Center of Biotechnology Information (NCBI), however, less than 5% are of environmental strains. The present study will fill the gap in genomic studies of environmental isolates of *A. pittii.*

The annotation of biosynthetic gene clusters (BGCs) in microbes provides important information about the distribution of various genes for secondary metabolite production, which can be useful for the identification of potent industrially relevant strains with novel and/or improved functionality (Dickschat, 2016). Furthermore, the use of genetic engineering and genome editing can be used to develop industrial-relevant strains for their wide applications. The microbe-produced secondary metabolites support the bacterial growth and development of the host as well as confer protection against infections (Osbourn, 2010). These BGCs include the NRPSs (nonribosomal peptide synthetases), PKSs (polyketide synthase), and RiPPs (ribosomally synthesized and post-translationally modified peptides) (Weber et al., 2015). Additionally, these BGCs explore information about the genes encoding key signature enzymes and antimicrobial proteins (AMPs) such as polymyxin, paenibacterin, and lipopeptides which showed strong inhibitory activity against bacteria, fungi and even cancer cells (Zhang and Galo, 2016; Liu et al., 2019). The use of AMPs in the food industry as natural antimicrobial agents is generally recognized as safe (GRAS), and promises safety and food product quality (Palmieri et al., 2016; Choyam et al., 2021). Considering these benefits, we explored the genome annotation of *Acinetobacter pittii* S-30 to identify BGCs and AMPs.

The carbohydrate-active enzymes (CAZymes) constitute a broader enzyme group that includes the glycosyltransferases (GTs), glycoside hydrolases (GHs), polysaccharide lyases (PLs), carbohydrate esterases (CEs), carbohydrate-binding modules (CBMs), and auxiliary activities (AAs) (Lombard et al., 2014). These are mainly involved in the degradation/rearrangements of glycosidic bonds in carbohydrates (Rytioja et al., 2014). However, the increasing industrial applications of CAZymes demand the exploration of new microbial strains for more diverse CAZymes.

The characterization of bacterial strains employing micromorphological, biochemical, growth pattern, and cellular chemotaxonomic characteristics is often in practice (Gharibzahedi et al., 2013), however, these strategies are not always adequate for the accurate classification and identification of newly isolated bacterial strains. The analysis of phenotypic similarity has defined many bacterial species, however, these methods are inadequate (Gevers et al., 2005), therefore, species definition and the best methods for taxonomic classification in prokaryotes continue to be debated (Bruen et al., 2006). The 16S rRNA gene sequencing has been widely used in bacteria to study phylogenetic relationships, however, this approach has certain limitations, as several environmental forces that configure the evolution of bacterial genomes act with different strengths on different parts of the genome and bacterial strains (Janda and Abbott, 2007; Chan et al., 2012).

To characterize the bacterial strains, robust sequencing technologies like “Next Generation Sequencing” provide valuable insight into the genome of organisms and allow the comprehensive analysis of genomic features (Roy et al., 2013). The functional annotation of genomic features can be utilized as a powerful tool for the development of genetically modified bacteria with improved functionality. Additionally, comparative genomics has emerged as a robust tool to identify and compare functionally important genomic elements (Wu et al., 2010). Therefore, in the present study, we focused on the genomic characterization of the environmental isolate *A. pittii* S-30 by whole genome sequencing (WGS) approach. Whole-genome analysis of this strain will provide opportunities to identify genes involved in stress protection, plant growth promotion (PGP), and genes involved in the production of secondary metabolites. The available WGS data of many *A. pittii* strains in the public database has been used for the comparative analysis. The strain possesses several beneficial gene features such as BGCs related to secondary metabolite production and presence of CAZymes, which equip the strain for use in the area of environmental biotechnology and other industrial applications. The present study is the first report demonstrating the in-depth genome and comparative genome characterization of an environmental isolate belonging to *A. pittii*.

## Materials and methods

### Characterization of the test isolate

The test isolate S-30, used in the present study, was isolated from the waste-contaminated site and identified by PCR-based method (Kumari et al., 2023). The strain was isolated from the waste contaminated soil sample collected from the Jhiri (23.40°N, 85.25°E), Ranchi, India. A test for starch hydrolysis, IMViC (Indole, Methyl Red, Voges–Proskauer, Citrate utilization), cellulase, pectinase, lipase, and catalase assay was performed following standard protocol (Harley and Prescott, 2002). To test the metal-resistance behavior, the test isolate was streaked on the LB-agar (Himedia, India) plate supplemented with different concentrations (1–5 mM) of metals such as copper sulfate (CuSO_4_), zinc sulfate (ZnSO_4_), mercuric chloride (HgCl_2_), cadmium chloride (CdCl_2_), and nickel sulfate hexahydrate (NiSO_4_.6H_2_O). The plates were incubated at 37°C for 24–48 h. Among the tested metals, S-30 showed the optimum growth on the ZnSO_4_ amended plate (up to 5 mM) and therefore, its growth profile was monitored in the liquid medium. To determine the growth pattern of the S-30, one loop of a single colony was inoculated in 100 ml of LB-broth medium supplemented with the above-mentioned heavy metals (5 mM concentration of each heavy metal) and grown in a shaker incubator at 37°C with 180 rpm for 24 h. The growth pattern was determined by using a spectrophotometer (HACH, USA) after a 4-h interval by measuring optical density (OD) at OD590. The strain was maintained in 20% glycerol in a −80°C freezer (Thermo Fisher Scientific, USA).

To check the antibiotic susceptibility, the disk diffusion method was used against different antibiotics such as erythromycin (15 μg), ampicillin (10 μg), kanamycin (30 μg), tetracycline (30 μg), ciprofloxacin (5 μg), gentamicin (10 μg), fluconazole (25 μg), streptomycin (10 μg), vancomycin (30 μg), and voriconazole (10 μg) as recommended by the Clinical and Laboratory Standards Institute (2012). These are the most common antibiotics used against both environmental and clinical strains. The KB009-HiCarbo Kit was used for carbohydrate fermentation tests as per manufacturer protocol. The antagonistic activity against bacterial pathogens such as *Bacillus subtilis, Salmonella typhi, Escherichia coli*, and *S. aureus* and fungal strains *Aspergillus niger, Microsporum gypseum*, and *Penicillium citrinum* was determined by the well diffusion method (Singh et al., 2015).

### Motility test

The culture was freshly grown on an LB-agar plate for a motility test. For the swimming test, culture was spot-inoculated on a solidified tryptone swim plate (1% tryptone, 0.5% NaCl, 0.3% agar) and incubated for 16 h at 25°C. For swarming, spot inoculation was performed on dextrose-containing swarming media (0.5% bacto-agar, 8 g L^–1^ nutrient broth, 5 g L^–1^ dextrose) and incubated for 24 h at 30°C. For twitching, LB agar (1% agar) media was used for stab inoculation, and inoculated plates were incubated at 30°C for 24–48 h. After incubation, the presence of a turbid circular zone indicated swimming, movement of inoculation for swarming, and a circular turbid zone for twitching activity (Connelly et al., 2004).

### Whole genome sequencing

The extracted genomic DNA was sequenced using a hybrid assembly approach on an Illumina MiSeq platform and the paired-end library was prepared using the NEB Next Ultra DNA Library Prep Kit. The Fast QC program was used for quality control of Illumina reads.^1^ The assembly was performed by Unicycler tool which first assembles short reads into an accurate and connected assembly and then uses long reads to produce a completed assembly. The initial contig level assembly was generated from Illumina short reads, followed by the use of nanopore long reads to build longer scaffolds by finding the overlap between generated contigs, which often allows it to resolve all repeats in the genome, resulting in a complete genome assembly. The transfer RNA (tRNA) and ribosomal RNA (rRNA) of the S-12 strain were identified using the tRNAscan-SE and RNAmmer (v1.2)^2^ software, respectively. The genome sequence was annotated by the RAST (Rapid Annotation using Subsystem Technology) annotation server (Aziz et al., 2008) to annotate the open reading frames, and BLAT v2.0 to validate the predictions. Cluster of orthologous (COG) functions of protein-coding sequences were determined using the RPS-BLAST algorithm for blast search against the COG database^3^ (Schäffer et al., 2001). The genes involved in metabolic pathways were annotated using Kyoto Encyclopedia of Genes and Genomes (KEGG) analysis tools (Kanehisa et al., 2016).

### Average nucleotide identity analysis

Average nucleotide identity (ANI) analysis was done to explore the genetic distance and relatedness for the genome sets containing S-30 and publicly available *A. pittii* genomes (Supplementary File 1) by ANI-BLAST (ANIb) using the software JSpecies v.1.2.1^4^ with a threshold of 95% as the cut-off for species (Richter and Rosselló-Móra, 2009). The occurrence of an ANIb value equal to or greater than 95% represents strains belonging to the same species (Lalucat et al., 2020). The ANI matrix was visualized using the tool Pyani (Pritchard, 2016). Additionally, we estimated *in silico* DNA–DNA hybridization using the Genome-to-Genome Distance Calculator program (Meier-Kolthoff et al., 2013).

### Antimicrobial and virulence analysis

To unravel the presence of AMR genes, the Comprehensive Antibiotic Resistance Database (CARD) (Alcock et al., 2020) was used using a homology-based approach (BLASTX) against the genome sequence of S-30 and the RESfinder (Bortolaia et al., 2020) database. For searching, BLAST output was filtered to a minimum of 80% identity and subject protein coverage. Similarly, the VFDB database^5^ was used against an assembled genome with criteria of a minimum of 80% identity using a homology-based approach (BLASTX) to identify the virulence genes (Chen et al., 2016).

### Prediction of biosynthetic gene clusters

The number and types of secondary metabolites BGCs in the genome sequence of *A. pittii* S-30 were identified by antiSMASH version 5.1.2 in combination with the Hidden Markov Model (HMM) to detect the BGCs-like region (Blin et al., 2021). Various unknown and characterized BGCs were identified and genetic similarities in gene clusters were predicted using antiSMASH 5.1.2.

### Prediction of carbohydrate–active enzyme

To unravel the presence of various CAZymes including GTs, GHs, PLs, CEs, AAs, and CBMs, the protein sequences of S-30 were annotated using the dbCAN2 server (Zhang et al., 2018), and BLAST-driven DIAMOND against the CAZy database (Lombard et al., 2014). The diversity of CAZymes in the closest relatives such as *A. pittii* strain 2014S06-099 (CP033540.1), *A. pittii* strain FDAARGOS 1216 (CP069586.1), *A. pittii* strain AP 882 (CP014477.1), *A. pittii* strain C54 (CP042364.1), *A. pittii* strain FDAARGOS 1215 (CP069504.1), *A. pittii* strain NQ-003 (CP035109.1), *A. pittii* strain AB17H194 (CP040911.1), *A. pittii* strain HUMV-6483 (CP021428.1), *A. pittii* strain ST220 (CP029610.1), *A. pittii* strain 2010C01-170 (CP029489.1), *A. pittii* strain 2014N21-145 (CP033568.1), *A. pittii* strain TCM (CP095407.1), *A. pittii* strain CEP14 (CP084921.1), *A. pittii* strain IEC338SC (CP015145.1), *A. pittii* strain 2012N21-164 (CP033535.1), *A. pittii* strain WCHAP100004 (CP027250.2), *A. pittii* strain WCHAP005069 (CP026089.2), *A. pittii* strain 2012N08-034 (CP033520.1), *A. pittii* strain AP2044 (CP087716.1), *A. pittii* strain YMC2010, *A. pittii* strain PHEA-2 (CP002177.1), *A. pittii* strain 2014N05-125 (CP033525.1), *A. pittii* strain 32292 (CP066124.1), *A. pittii* strain FDAARGOS_1217 (CP069496.1), *A. pittii* strain FDAARGOS_1214 (CP069537.1), *A. pittii* strain WCHAP005046 (CP028574.2), *A. pittii* strain JXA13 (CP054137.1), *A. pittii* strain AP43 (CP043052.1), *A. pittii* strain 2014S07-126 (CP033530.1), *A. pittii* strain Ap-W20 CP027658.1, *A. pittii* strain A1254 (CP049806.1), *A. pittii* strain AbW39 (CP029005.1), *A. pittii* strain XJ88 (CP018909.1), and *A. pittii* strain WCHAP100020 (CP027254.3) was evaluated.

### Analysis of genetic diversity and recombination in *A. pittii* strains

A core genome tree was constructed with non-recombinant genes by performing a BlastP search with an e-value cut-off of 1.0e−15 among *A. pittii* strains. Core genes were selected based on alignment coverage of ≥70%, following the approach described in the *Staphylococcus* and *Bifidobacterium* genus (Graña-Miraglia et al., 2018; Deb, 2022). To eliminate recombinant genes, the PhiTest feature from the PhiPack program was employed (Bruen et al., 2006). The resulting set of non-recombinant core genes was concatenated and used to build a maximum-likelihood tree using the RAxML tool with a generalized time-reversible (GTR) model (Stamatakis, 2014). We also aimed to compare a range of recombination parameters and calculate correlation profiles among *A. pittii* genomes. In pursuit of this goal, we employed the core genome alignment as input for the mcorr tool (Lin and Kussell, 2019).

### Comparative genome analysis

The analysis of orthologous gene clusters was performed using the Orthovenn2 program (Xu et al., 2019) with default parameters using the S-30 and it is closely related *A. pittii* strains. The strains that showed the highest similarity (>99%) in ANI, were used for analysis. The circular genome comparison of the draft assembly genome of S-30 was performed against the reference genome using the BRIG (Blast Ring Image Generator) Tool (Alikhan et al., 2011). BLAST was performed on nine *Acinetobacter* genomes which were constructed using NCBI local BLAST-2.10.1. Genomic islands (GIs) were predicted using Island Viewer 4 (Bertelli et al., 2017). Prophages were found using PHAST (Arndt et al., 2019). Clustered regularly interspaced short palindromic repeat sequences (CRISPRs) were found by the CRISPR Finder (Grissa et al., 2007).

### Data deposition

The genome sequence of S-30 is available at NCBI with the BioProject PRJNA934688, Biosample SAMN33277265, and Genbank accession no. JARJDL000000000.

## Results

### Characterization of test isolate S-30

The test isolate used in the present study was identified as *A. pittii* S-30. The isolate showed a negative result for IMViC, pectinase, and positive result for amylase, lipase, cellulase, and catalase tests (Supplementary Table 1). The growth kinetics pattern clearly illustrates that isolate showed the higher growth capacity at 5 mM of ZnSO_4_ stress as compared to other tested heavy metals (Supplementary Figure 1A). Among the various carbon sources, strain was able to utilize lactose, xylose, maltose, galactose, sucrose, raffinose, melibiose, sorbitol, glycerol, Inositol, Inulin, D-arabinose, citrate, malonate, ONPG, and mannoside (Supplementary Table 1). It showed higher sensitivity (20–25 mm) against ampicillin, kanamycin, tetracycline, gentamicin, ciprofloxacin, vancomycin, and moderate sensitivity (12–18 mm) to streptomycin, and fluconazole. The strain was found to be resistant to voriconazole and erythromycin. The test isolate showed good antagonistic activity against *S. typhi, E. coli*, and moderate against *B. subtilis* and *S. aureus.* Against the tested fungal strains, strain showed good activity against *A. niger, M. gypseum*, and moderate activity against *H. gypsium*, and *P. citrinum* (Supplementary Table 2). S-30 showed swimming, swarming, and twitching motility (Supplementary Figure 1B).

### Genome analysis

**FIGURE 1:**
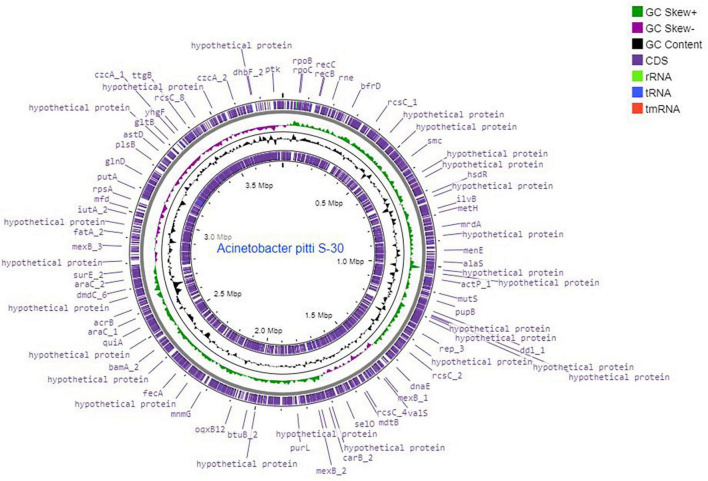
Circular genome map of *A. pittii* S-30 constructed by DNA plotter. Rings from inside represent the following: (1) CDS, (2) tRNA, (3) rRNA, (4) tmRNA, (5) GC content, (6) GC skew^+^, (7) GC skew^–^, (8) position labels for genome length (Mbp).

A total of 6.10 million sequencing reads were generated for the strain S-30. Hybrid assembly was performed using the Unicycler v-0.48 tool for the assembly of a single contig of 3.78 Mb size (Figure 1). The assembly was validated using the NCBI-NR Blast program which showed the maximum homology with other *A. pittii* strains. The overall average G + C content of S-30 is 38.7% (Table 1). A total of 62 RNAs were annotated in the S-30 genome. Further, gene and protein prediction from the draft genome using the Prokka v1.14 tool identified a total of 3,516 protein-coding genes.

**TABLE 1:** General features of *A. pittii* S-30 genome.

Property	Term
Geographical location	23.40°N, 85.25°E
Sample collection	Soil
NCBI bioproject ID	PRJNA934688
Biosample ID	SAMN33277265
GenBank ID	JARJDL000000000
Sequencing platform	Illumina HiSeq 2000
Genome size	3.78 Mb
G + C Content	38.7%
Protein-coding genes	3,516
RNAs genes	62

### ANI and core genome phylogeny

**FIGURE 2:**
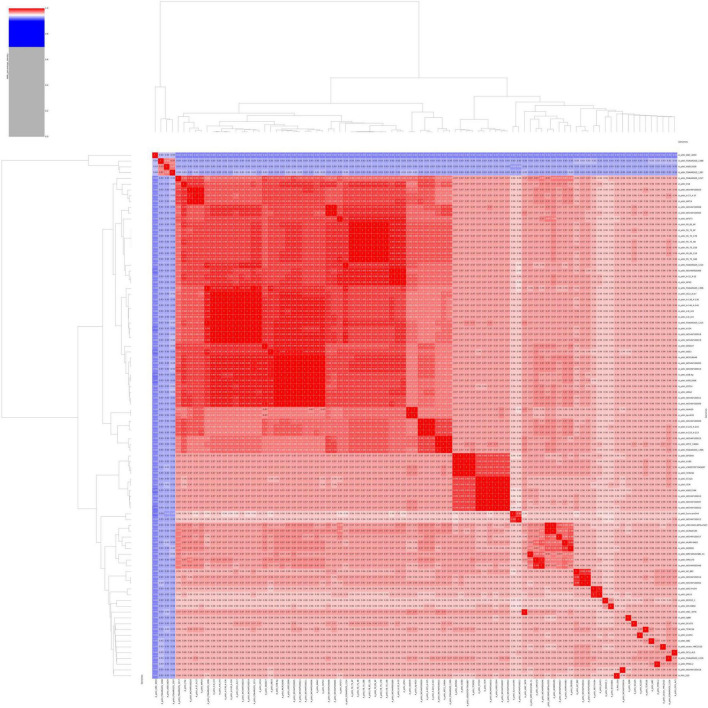
An average nucleotide identity (ANI) matrix was constructed to illustrate genomic relatedness through a multi-genome comparison among *A. pittii* strains retrieved from the NCBI database.

To re-evaluate the phylogenetic relationship of S-30 within the *Acinetobacter* genus, the ANI percentage of the S-30 genome was calculated with respect to other sequenced *A. pittii* strains, available at the NCBI database. The statistics of downloaded *A. pittii* genomes were estimated, the genome sizes and GC contents ranged from 3.7 to 4.3 Mb and 38.61% to 39.97%, respectively (Supplementary File 2). The genomes code in between 3,625 and 4,331 genes, and 28–78 tRNAs (Supplementary File 2). The assessment of genomes sourced from the NCBI database included an evaluation of their quality and completeness (Supplementary File 3). Only genomes deemed to be of high quality, with a completeness exceeding 90% and contamination below 3%, were eligible for subsequent downstream analysis. The genome of *A. pittii* strains showing >95% BLAST percentage similarity between *A. pittii* S-30 and various reference *A. pittii* strains, excluding *A. pittii* ANC4050, *A. pittii* FDAARGOS 1398, *A. pittii* AS012629, and *A. pittii* FDAARGOS 1397, which has been summarized in the ANI matrix (Figure 2). These findings provide strong evidence for the *A. pittii* species classification of our S-30 isolate. However, the lower ANI values exhibited by four *A. pittii* strains indicate uncertainty in their taxonomic placement within this species group. Additionally, it has been observed that the *in silico* DDH (DNA–DNA hybridization) values of the taxonomically disputed *A. pittii* strains were below 70%, which is in accordance with the ANI findings and provides support for the hypothesis that those strains do not belong to the *A. pittii* species group (Supplementary File 4).

The aforementioned observation, supported by ANI and *in silico* DDH analysis, indicates that four strains were erroneously labeled as *A. pittii* species. These strains require additional taxonomic validation before being classified as *A. pittii* species. We conducted an analysis of the core genomes within the *A. pittii* species to explore the phylogenetic relationships and genetic diversity among the strains. Using the concatenated nucleotide sequences of 1,704 single-copy non-recombinant ortholog core genes found in all 90 genomes, a core genome tree was constructed in order to elucidate the evolutionary connections among the bacterial strains. The core genome phylogenetic tree has demonstrated a number of distinct genomic clusters, indicating genetic similarity varies with different phylogenetic clades of the *A. pittii* strains. *A. pittii* strains isolated from various hosts and environmental settings were clustered independently, with no distinct clade based on isolation source, geographical location and host type (Supplementary Figure 2). This suggests that host or environmental selection pressure may not have been the prevailing factor driving the evolution of globally circulating *A. pittii* strains.

### RAST functional annotation

**FIGURE 3:**
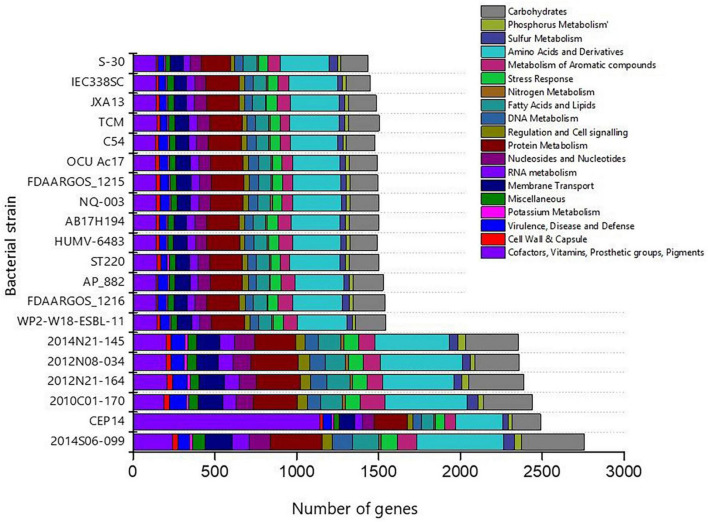
Bar diagram demonstrating the distribution of gene functional categories in the closely related gnome of *A. pittii.* The closely related genomes of *A. pittii* were downloaded from NCBI database.

The genome sequence of S-30 was further annotated by RAST, and subsystem coverage and non-subsystem coverage generated are 31% and 69%, respectively (Supplementary Figure 3A). RAST analysis predicted the subsystem category distribution (Supplementary Figure 3B) and subsystem feature counts (Supplementary Figure 3C). The top three subsystem features of S-30 are amino acids derivatives (297 genes), protein metabolism (179 genes), carbohydrate metabolism (167 genes), followed by cofactors and vitamins (139 genes). The other subsystems include the membrane transporter and fatty acid metabolism (84 genes), metabolism of aromatic compounds (77 genes), nucleotide synthesis (69 genes), and stress responses (58 genes). The various genes related to stress responses (Supplementary Table 3), phosphate metabolism (Supplementary Table 4), sulfur metabolism (Supplementary Table 5), and the metabolism of aromatic compounds (Supplementary Table 6) were identified. Additionally, we did a comparative assessment of the distribution of various genes in the closely related *A. pittii* strains which illustrated the higher number of genes in *A. pittii* strain 2014S06-099 (CP033540.1), followed by *A. pittii* strain CEP14 (CP084921.1), *A. pittii* strain 2010C01-170 (CP029489.1), *A. pittii* strain 2012N21-164 (CP033535.1). An equal distribution of genes was observed in the *A. pittii* strain 2014N21-145 (CP033568.1) and *A. pittii* strain 2012N08-034 (CP033520.1) (Figure 3). The strain *A. pittii* strain 2014N21-145 showed the higher number of genes for protein metabolism (289), amino acids and derivatives (501), potassium metabolism (21), whereas *A. pittii* strain 2012N08-034, higher gene numbers were recoded for membrane transport (143), nucleosides and nucleotides (124), phosphorus (45), and carbohydrate metabolism (324).

### COG and KEGG analysis

**FIGURE 4:**
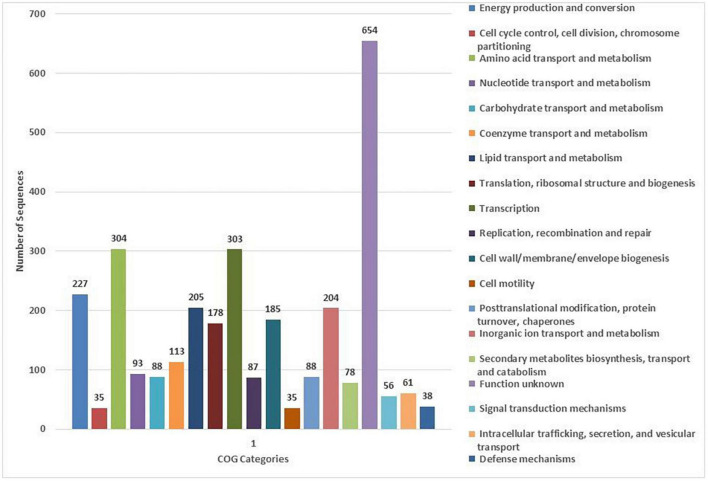
Classification of the cluster of orthologous (COG) functional annotation of the *A. pittii* S-30 genome. Colored bars indicate the number of genes assigned to each COG functional category.

**FIGURE 5:**
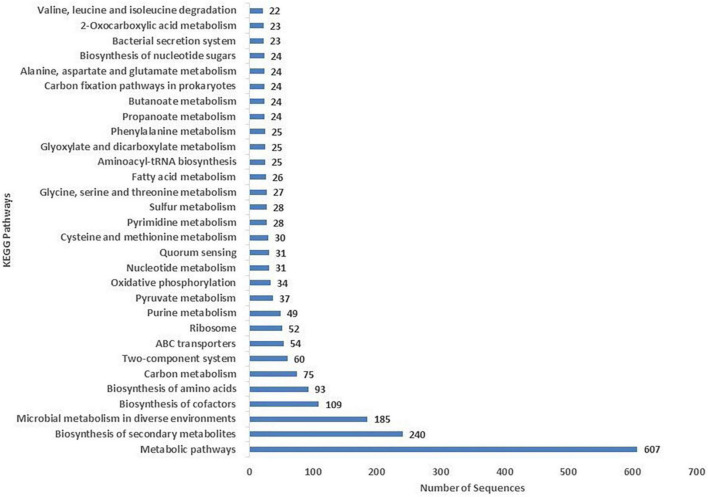
Kyoto Encyclopedia of Genes and Genomes (KEGG) were utilized for the retrieval of metabolic pathways of S-30 genes. KAAS server at KEGG database used for genes functional annotation by BLAST comparison with the manually curated database of KEGG-GENES.

The COG database was used for the functional classification of predicted genes, whose distribution within the COG categories is provided in Figure 4. The highest number of genes (654) were identified with unknown functionality. Furthermore, analysis observed 304 genes associated with amino acid metabolism, 303 with transcription processes, 227 genes with energy production processes, 205 with lipid transport and metabolism, 204 with ion transport and metabolism, 185 with cell-wall biogenesis, and 178 with ribosome structures and biogenesis, respectively. KEGG analysis identified the genes belonging to the various metabolic pathways. The highest number 607 was recorded for genes involved in different metabolic pathways, 240 with the biosynthesis of secondary metabolites, 185 with microbial metabolisms, 109 with biosynthesis of cofactors, 93 with biosynthesis of amino acids, and 75 with carbon metabolism (Figure 5). Similarly, 60 were associated with two-component systems, 54 with ABC-transporters, 52 with ribosome biogenesis, 49 and 47 with purine and pyruvate metabolism, respectively.

### Gene ontology

**FIGURE 6:**
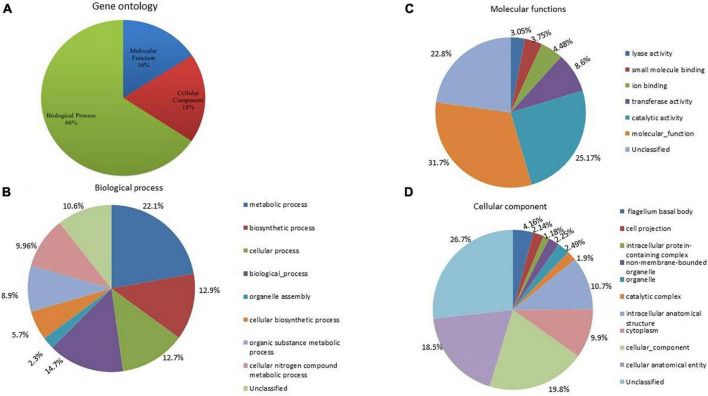
**(A)** Gene ontology analysis of *A. pittii* S-30 genome, the genome was annotated to unravel the genes involved in **(B)** biological process, **(C)** molecular functions, and **(D)** cellular component.

To assign the functional classification of proteins, a gene ontology was performed. A total of 66% of genes were related to biological processes, 16% to molecular functions, and 18% to cellular components (Figure 6A). Among the biological processes, 22.1% were related to metabolic processes, 14.7% to various biological processes, 12.9% to biosynthetic processes and cellular processes, 9.96% to cellular and organic compound metabolic processes, and 5.7% to cellular biosynthetic processes (Figure 6B). A total of 12.7% were identified as unclassified functions. In the cellular component, 19.8% were related to the cellular component, 18.5% to the cellular anatomical entity, 10.7% to the intracellular anatomical structure, 10% to the cytoplasm, and 4% to the bacterial flagellum structure (Figure 6C). Approximately, 26.7% of genes were annotated as having unclassified functions. In molecular processes, 31.7% of genes were related to molecular functions, 25.17% had catalase activity, 8.6% had transferase activity, 4.9% had ion-binding activity, 3% had lyase activity, and 22.8% were unclassified (Figure 6D).

### AMRs and VFDB

Comprehensive Antibiotic Resistance Database analysis identified the different classes of AMRs which have been summarized in Supplementary Figure 4. A number of resistance genes harbored within the genomes of the S-30 strain were identified and gene families associated with resistance-nodulation-cell division (RND) antibiotic efflux pump, major facilitator superfamily (MFS) antibiotic efflux pump, beta-lactamase, ATP-binding cassette (ABC) antibiotic efflux pump, multidrug and toxic compound extrusion (MATE) transporter, and pmr phosphoethanolamine transferase were identified. Additionally, we observed the genes related to fluoroquinolone resistance (gyrA), daptomycin resistance (pgsA), pyrazinamide resistance (pncA), elfamycin resistance (EF-Tu), streptothricin acetyltransferase (SAT), and rifampin glycosyltransferase (Supplementary File 5).

VFDB analysis identified the various genes belonging to the virulence class such as adherence, biofilm formation, enzymes, immune evasion, iron-uptake, efflux pumps, endotoxins, secretion systems, and toxin production (Supplementary File 6). In the adherence, genes associated with categories like LPS (orfH, lpxABCD), flagella (flmH), and pilus (csgF) were observed. The various genes associated with biofilm formation (biofilm-associated protein: bap; csu pili operon: csuABCDEF; polysaccharide poly-N-acetylglucosamine: pgaABCD), enzymes (phospholipase: plcC, plcD), iron uptake (acinetobactin: basABCD; barAB, bauABCDEF, enterobactin synthesis: entABCD; heme biosynthesis: hemCEGH), efflux pumps (acrB), toxins (hemolysin: hlyA), stress adaption (catalase: katA; manganese transport system: mntB), and flagellar assembly (flgB, flgC, flgD, flgE, flgF, flgG, flgH, flgL, flgK, flgL, flgM, flhA, flhB, flhC, flhD, fliA, fliD, fliE, fliF, fliG, fliH, fliJ, fliK, fliL) were observed (Supplementary File 6). Additionally, a gene associated with type VI secretion systems (clpV) was also noted.

### BGCs analysis

The S-30 genome was annotated using the antiSMASH database to identify the secondary metabolite pathways. A total of eight gene clusters were identified including arylpolyene, non-ribosomal polypeptides (NRPS) like the betalactone cluster, hserlactone, redox-cofactor, and NRP-metallophore. The four NRPS clusters found in S-30 showed 100% similarity to 2-amino-4-methoxy-*trans*-3-butenoic acid, pyoluteorin, azetidomonamide, and pseudopaline, respectively (Table 2).

**TABLE 2:** The identified BGCs genes in the *A. pittii* S-30 genome.

Cluster	Type	From	To	Most similar known cluster	Similarity (%)
1	Arylpolyene	299,716	342,714	Other	45
2	Arylpolyene	594,975	636,204	RiPP	16
3	RiPP-like	947,227	959,425	–	–
4	Betalactone	306,099	1,335,178	NRP	13
5	NRP-metallophore	1,976,847	2,030,049	NRP	100
6	NI-siderophore	2,392,409	2,408,836	Other	16
7	Redox-cofactor	2,860,010	2,882,200	NRP + polyketide	13
8	NRPS	3,648,789	3,692,748	Polyketide	4

### CAZymes analysis

**FIGURE 7:**
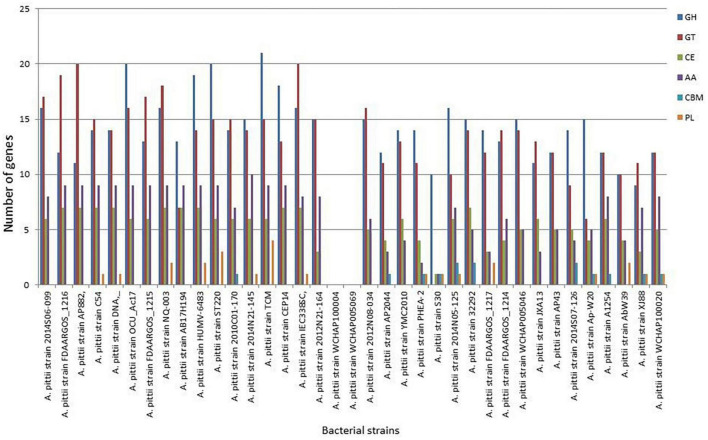
The distribution of various CAZymes like carbohydrate-binding modules (CBMs), glycoside hydrolases (GHs), glycosyltransferases (GTs), polysaccharide lyases (PLs), carbohydrate esterases (CEs), and auxiliary activities (AAs) in *A. pittii* S-30, and their comparison to other closely related *A. pittii* strains.

To unravel the enzymes involved in the breakdown of complex carbohydrates, the S-30 genome was analyzed by the dbCAN2 server. As a result, 14 CAZymes genes were identified in the S-30 genome, which were classified into GHs, GTs, CBMs, CEs, and AAs. Of these, the most abundant CAZymes were GHs (10), followed by PLs (1 gene), CBMs (1 gene), AAs (1 gene), and CEs (1 gene) (Figure 7). The different groups of CAZymes were also compared to other genomes of *A. pittii* strains and a comparison of different CAZymes has been demonstrated in Figure 7. Among the compared genomes, a higher number of CAZymes (55) was observed for *A. pittii* strain TCM (CP095407.1), followed by *A. pittii* strain ST220 (CP029610.1, 53 CAZymes), and *A. pittii* strain IEC338SC (CP015145.1, 52 CAZymes), respectively (Figure 7).

### Analysis of genetic diversity and recombination in *A. pittii* strains

The genetic diversity analysis revealed information about the population dynamics of *A. pittii* strains. The number of segregation sites and genetic diversity in the sequences were quantified in Tajima’s D (11). A higher frequency of rare alleles, which may be due to the selection sweep or population expansion, leads to a negative Tajima’s D value. Our analysis revealed that Tajima’s D values are negative along the core genomic region, indicating the probable population expansion of *A. pittii* strains across the globe (Supplementary File 7).

**FIGURE 8:**
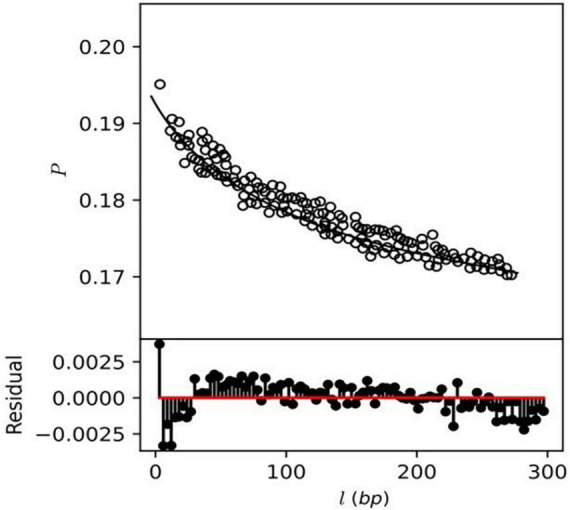
Correlation profiles of recombination (depicted as circles) were estimated from the genomes of *A. pittii* strains.

This study also investigates the homologous recombination rate among the *A. pittii* strain using the mcorr method, which produces a correlation profile with pairwise genome comparison to determine the likelihood that two genomes differ at one site if differences exist at another locus (14). The correlation profile *P (l)* of core genes showed monotonic decay as a function of locus distance (*l*) exhibiting recombination in *A. pittii* strains (Figure 8), while a constant or flat correlation profile would denote an absence of recombination. A total of five recombination parameters, which include sample diversity (d), mutational divergence (θ), recombinational divergence (ϕ), relative rate of recombination to mutation (γ/μ), and recombination coverage (c) were obtained from the recombination analysis of *A. pittii* strains (Supplementary File 8). The d value or sample diversity in *A. pittii* species is 0.5 which estimates from both recombination and mutations contributing to clonal evolution. This indicates *A. pittii* is experiencing a high sample diversity compared to other pathogenic bacterial species *Yersinia pestis* (*d* = 0.0091), *P. aeruginosa* (*d* = 0.027), and *K. pneumoniae* (*d* = 0.13). The remaining four recombinant parameters identified as θ, ϕ, γ/μ in *A. pittii* strains had values of 1.3, 9.5, 7.2, and 0.9, respectively. The list of non-recombinant genes is mentioned in Supplementary File 9.

### Comparative genome and pangenome analysis

The core genome was used for comparison between S-30 and the other closely related *A. pittii* strains. The observed analysis suggests that three genes were found to be shared by strains *A. pittii* strain 2014S06-099, *A. pittii* strain FDAARGOS 1216, *A. pittii* strain AP 882, *A. pittii* strain C54 and WP2-W18-ESBL-11 (Supplementary Figure 5A). The first pattern shows the formation of gene clusters, second pattern shows cluster counts and the third pattern represented in the form of a stacker bar shows the total protein counts (Supplementary Figure 5B). To highlight the overlapping gene clusters, a pairwise heatmap was performed for S-30 and other strains. A red color gradient showing the highest overlapping gene cluster thresholds was noted between *P. aeruginosa* S-8 and *P. aeruginosa* W16407 (Supplementary Figure 5C). A circular comparison performed by BRIG revealed the overall genome of *A. pittii* S-30 has a high degree of sequence similarity (>99%) with other compared genomes (Supplementary Figure 6). GIs regions in the genome demonstrate horizontal gene transfer (HGT) events and might be involved in important functions such as symbiosis or pathogenesis (Supplementary Figure 7). In S-30 GIs regions, we observed the presence of phytanoyl-CoA-dioxygenase, alpha/beta hydrolases, aminotransferases, transcriptional regulators (TetR, IcIR, and LysR family), aminoglycoside, and phosphotransferase. Several genes in GIs were identified as hypothetical proteins with unknown functions.

## Discussion

Here, in the present study, we report the detailed characterization of *A. pittii* S-30, isolated from the waste-contaminated soil sample. The phenotypic characterization and antibiotic susceptibility patterns are insufficient to provide insight into the various gene repositories for different characteristics, therefore, we did a detailed genomic and comparative genome analysis of S-30. The genome analysis of bacteria inhabiting extreme conditions provides an opportunity to unravel the mechanisms involved in survival (Baquero et al., 2021). Similarly, comparative genome analysis provides important information of relevant traits between functionally important genetic elements constituting the core genome (Sinha and Krishnan, 2021). The establishment of an accurate phylogenetic tree and ANI analysis supports the understanding of the major transition events in evolution (Emms and Kelly, 2015; Kapli et al., 2020), and is useful for calculating the genomic distance, which could be helpful to overcome the challenges caused by HGT and mutation events (Konstantinidis and Tiedje, 2007). The use of ANI and dDDH values to reidentify the bacterial species has been previously proven to be effective (Morimoto et al., 2020). Our study supports this approach and emphasizes the importance of techniques that enable the use of these values to more accurately classify *Acinetobacter* isolates.

### Osmotic stress adaptation and metal-resistance genes

Genome annotation identified the various genes related to stress responses including glutathione S-transferase, metallothionein, superoxide dismutase, thioredoxin peroxidase, catalase, iron-binding ferritin-like antioxidant protein, ferroxidase, zinc uptake regulation protein (ZUR), and ferric uptake regulation protein (FUR). In addition, the various transcriptional regulators belonging to nitrite-sensitive transcriptional repressor (NsrR), redox-sensitive transcriptional activator (SoxR), fumarate and nitrate reduction regulatory protein (Fnr), and FUR family of transcriptional regulator were observed. To protect against metal stress, genes related to cobalt-zinc-cadmium resistance protein (CzcD), copper homeostasis protein (CutF), copper chaperone (CopZ), zinc transporter (ZitB), nickel-cobalt-cadmium resistance protein (NccA), and chromate resistance protein (ChrB) were identified in S-30 genome. Various efflux pumps related to heavy metal RND efflux outer membrane protein, multidrug and toxin extrusion (MATE) family efflux pump YdhE/NorM, cobalt/zinc/cadmium efflux RND transporter, cation efflux system protein, cadmium-transporting ATPase, magnesium and cobalt efflux protein CorC, and AcrR family of multidrug efflux pumps were noted.

### Antibiotic and virulence genes

The genomic analysis of strain S-30 resulted in the identification of a number of antibiotic-resistant genes (ARGs). The detailed genomic analysis revealed many ARGs, specifically against ampicillin, tetracycline, erythromycin, penicillin, transporters, and drug targets. One major mechanism underpinning microbial resistance against antibiotics is by effluxing out these compounds once encountered by the cellular environment (Blanco et al., 2016; Buffet-Bataillon et al., 2016). The genomes of *A. pittii* were examined for antibiotic-resistant genes, revealing that the antibiotic efflux pump genes (abeS and abeM), as well as the RND-type efflux pump genes (AdeIJK and AdeFGH), are the most prevalent resistance genes consistently identified among the strains (Supplementary Table 4) (Su et al., 2005; Srinivasan et al., 2009). This finding coincides with the drug-resistant profile of clinical *A. pittii* isolates (Chopjitt et al., 2021). It is widely recognized that bacteria can acquire drug resistance phenotypes through gene acquisition facilitated by diverse mechanisms of gene transfer events (Sun et al., 2019; Evans et al., 2020). Earlier research has documented the transmission of drug resistance genes from livestock farms to *Acinetobacter* species in the environment (Wang and Sun, 2015). Moreover, previous investigations have highlighted the presence of the Acb complex, which consists of four *Acinetobacter* species (*A. calcoaceticus, A. baumannii*, *A. pittii*, and *A. nosocomialis*) (Gerner-Smidt et al., 1991; Nemec et al., 2011). This complex lends support to the concept of potential transfer events of resistant genes (abeS, abeM, AdeIJK, and AdeFGH) from *A. baumannii* species to the circulating *A. pittii* strains (Su et al., 2005; Srinivasan et al., 2009; Chopjitt et al., 2021).

Additionally, *blc, bli, blR*, and *blaR* resistance genes exist in the genome of the S-30 for resistance to β-lactam group antibiotics, including penicillin, cephalosporin, cephamycin, and carbapenem, supply multi-resistance by breaking the antibiotics’ structure. S-30 genome carries β-lactam resistance genes that are responsible for penicillin binding, hydrolase and regulation to have become effective at their function as antibiotic resistance enzymes (Massova and Mobashery, 1998). Earlier, it was reported that heavy metal pollution increases the metal resistance and reduce antibiotic sensitivity due to co-regulation of genes (Baker-Austin et al., 2006; Seiler and Berendonk, 2012). Hence, we assume that the isolation of S-30 from the heavy metal region promoted heavy metal and antibiotic resistance as well.

The S-30 strain showed the presence of virulence genes involved in adhesion, recognition, and degradation processes which increase the pathogenicity of the bacteria (Zhu et al., 2015). These virulence factors enable the bacteria to invade the host atrociously, e.g., chitinases possess the ability to degrade the chitin present on the exoskeleton of insects, whereas bacillolysin enables the hydrolysis of amino leucine and phenylalanine that elicit the innate immune system (Nielsen-Leroux et al., 2012). Genes associated with collagenases and phospholipases are associated with the disruption of the intestine and midgut epithelial cells that help the bacterium colonize inside the host cells (Schwan et al., 1994; Peng et al., 2016).

### Stress protection

Under stress conditions, the generated reactive oxygen species (ROS) severely damage to cell and it may lead lysis of cells. To cope with this situation, cells usually synthesize antioxidant enzymes and antioxidant substances to reduce the level and damage caused by ROS. In the OS-1 genome, antioxidant enzymes such as peroxidases (POD) (one gene), catalases (CAT) (three genes), superoxide dismutase (SOD) (three genes), and thioredoxin (two genes) were identified. The main antioxidant is glutathione and the genes related to glutathione synthetase (two genes), glutathione reductase (one gene), glutathione peroxidase (two genes), and glutathione S-transferase (nine genes) were noted.

In response to abiotic stressors (salt, alkali, drought, and temperature stress), bacteria synthesize various osmotic protectants. Among osmoprotectants, proline is considered as strong hydrophilic and highest solubility, which help to prevent cell dehydration (El Moukhtari et al., 2020). The genes that are required for proline synthesis, glutamate kinase and pyrroline-5-carboxylic acid reductase, were found in strain S-30 genome. Similarly, osmoprotectant belonging to the carbohydrate’s family such as the synthesis of trehalose (*glgABC* and *TreYZ*), starch synthase, 1,4-alpha-glucan branching enzyme, and malto-oligosyltrehalose trehalohydrolase were identified in the S-30 genome. Another osmoprotective agent “betaine” belongs to the alkaloids family and gene (*betB*) encoding betaine-aldehyde dehydrogenase related to betaine synthesis in the S-30 genome was observed.

The extracellular secreted polysaccharides can also promote biofilm formation and play an important role in alleviating abiotic stressors (Morcillo and Manzanera, 2021). Genome annotation analysis showed that strain S-30 possesses UTP-glucose-1-phosphate uridylyltransferase and mannose-1-phosphate guanylyltransferase which are responsible for the synthesis of UDP-glucose and UDP-mannose, respectively. Additionally, 11 genes encoding glycosyltransferase were identified in the genome of S-30, which play an important role in the assembly of polysaccharides. This indicates that strain S-30 has the ability to synthesize polysaccharides, and the corresponding polysaccharide species may be different from those of other species of *Acinetobacter.* Moreover, two-component systems (TCS) protect the bacterial cells from adverse environmental effects by producing antioxidant enzymes, extracellular polysaccharides, proline, and trehalose. In addition, in response to biotic infections, TCS enables the host to produce oxidative stress reactions such as hydrogen peroxide production to resist infection (Xikeranmu et al., 2020).

### Chemotaxis and motility

The bacterial chemotaxis consists of a series of proteins such as methyl-accepting chemotaxis proteins (Mcps), scaffold proteins (CheW/CheV), histidine kinase (CheA), reaction regulator protein (CheY), methylesterases (CheB and CheR), phosphatases (CheC/CheX/CheZ), and deamidase (CheD) with different functions. The S-30 genome contained 21 chemotaxis-related genes, including 8 genes encoding chemoreceptor (McpA), as well as 11 genes encoding chemotactic protein. The chemotactic proteins included CheA, CheB, CheD, CheR, CheW, and CheY. In addition, eight genes related to flagellar assembly encoding FliF, FliG, FliH, FliN, FliL, MotA, and MotB (Morimoto and Minamino, 2014) were detected in S-30 genome. In the vicinity of scaffold protein CheW/V, Mcps links with CheA, which is activated to phosphorylate downstream CheY (Boukhvalova et al., 2002). The phosphorylated CheY-P then interacts with the flagellar-motor complex through the flagellin FliM to regulate the direction of bacterial movement (Welch et al., 1993). The phosphatases CheC/CheX/CheZ dephosphorylate the CheY-P, thereby allowing the CheY to continuously accept phosphate groups (Sircar et al., 2013). The deamidase CheD regulates the phosphatase activity of CheC (Chao et al., 2006). The presence of a large number of chemotactic proteins endows strain S-30 with a strong potential for colonization.

### Metabolism

The genome of S-30 consists mostly of known genes encoding various metabolic modules and pathways that support its growth. The organic acids secreted by bacteria, e.g., citric acid, pyruvic acid, acetic acid, gluconic acid, oxalic acid, and malic acid enhance the dissolution of inorganic phosphorus, whereas organophosphorus mineralization mainly depends on enzymatic hydrolysis, including phosphatase, phytase, and C-P lyase (Bunemann, 2015). Genome annotation showed that strain S-30 possesses various enzymes which can release both inorganic and organic phosphorus from the degradation of complex polymeric compounds. Strain S-30 just as many bacteria have genetic capacity for sulfur, phosphorus, and different carbohydrate metabolisms. The S-30 sulfur metabolism pathway is quite complex and encoded by *soxABXYZDFCRSWH* gene cluster. The sulfur oxidation requires only five *sox* genes whose products form three key periplasmic protein complexes: *soxYZ*, a sulfur carrier protein, *soxXA*, a c-type cytochrome complex, and *soxB*, a sulfate thiol hydrolase (Janssen et al., 2010). On the other hand, sulfur assimilation involves the *ssuA*—alkanesulfonates-binding, *ssuB*—alkanesulfonate ABC transporter ATP binding, s*suF*—organosulfonate utilization, *ssuC*—alkanesulfonates transport system permease, and *ssuD*—alkanesulfonate monooxygenase proteins. In S-30 genome, several operons responsible for carbohydrate metabolism such as maltose and maltodextrin utilization (*malEFGKMPRAZ*) and mannose metabolism (*manYZBCEFGKL, mtpEFGKL*) were identified.

### BGCs analysis

Region 1 represented the presence of acyl carrier protein, ribosome biogenesis proteins, the efflux transport system, and outer membrane factor (OMF) lipoproteins. The acyl carrier protein (ACP) acts as a cofactor in both fatty acid and polyketide biosynthesis (Cronan John, 2014). Ribosome biogenesis proteins involve the coordinated function of over 200 proteins in the synthesis and processing of the three prokaryotic or four eukaryotic rRNAs, assembly of rRNAs with the ribosomal proteins, and fall into various energy-consuming enzyme families including ATP-dependent RNA helicases, AAA-ATPases, GTPases, and kinases (Kressler et al., 2009). In region 2, we observed the genes belonged to LysR family of transcriptional regulator (LTTRs), translation elongation factor G, and thioredoxin reductase. LTTRs are the most common type of prokaryotic DNA-binding protein, and are composed of an N-terminal helix–turn–helix DNA-binding domain and a C-terminal co-inducer binding domain (Maddocks and Oyston, 2008). LTTRs are known as regulatory proteins which can function as either activators or repressors of gene expression (Schell, 1993). In bacteria, LTTRs control genes related to virulence (Doty et al., 1993; Russell et al., 2004), quorum sensing (O’Grady et al., 2011), motility (Heroven and Dersch, 2006), and metabolism (Hartmann et al., 2013). In region 3, genes related to vanillate transporter (VanK), short-chain dehydrogenase/reductase family, and lipase/esterase were noted.

Region 4, represented the genes for hydroxymethylglutaryl (HMG)-CoA lyase, methylglutaconyl (MG)-CoA hydratase, methylcrotonyl-CoA carboxylase (MCC), and LysR family of transcriptional regulators. Hydroxymethylglutaryl (HMG)-CoA lyase catalyzes the final step of leucine degradation and plays a key role in ketone body formation (Luo et al., 2017). MG-CoA hydratase belongs to the enoyl-CoA hydratase/isomerase superfamily, which has been observed to be involved in the regulation of metabolic enzymatic activity (Mack et al., 2006). MCC is a biotin-dependent carboxylase that catalyzes the regulatory step in the leucine catabolic pathway (Lehnert et al., 1996). Members of this family have three structurally conserved functional domains; the biotin carboxyl carrier domain, which carries the biotin prosthetic group; the biotin carboxylation domain, which catalyzes the carboxylation of biotin; and the carboxyltransferase domain, which catalyzes the transfer of a carboxyl group from carboxybiotin to the organic substrate specific for each carboxylase (Abu-Elheiga et al., 1995).

The fifth cluster contained genes for the iron transport protein, the ABC uptake transporter protein, the siderophore biosynthesis protein, and the AcrR family of transcriptional regulators. ABC transporter proteins consist of multiple subunits, including transmembrane proteins and one or two of which are membrane-associated AAA ATPases, which utilize the energy of adenosine triphosphate (ATP) binding and hydrolysis to provide the energy needed for the translocation of substrates across membranes (Davidson et al., 2008). AcrR has a strong affinity for DNA and is a well-characterized functional protein of the transcriptional regulation system that confers resistance to the antibiotic tetracycline (Colclough et al., 2019). It binds to the operator site and represses the transcription of its own gene (Deng et al., 2013).

Region 6th BGC represented the genes belonging to the siderophore synthetase, 2,3-diaminopropionate for siderophore biosynthesis proteins SbnA/SbnB, and outer membrane Fe transport proteins. Siderophore synthetases are involved in the production of other siderophores including desferrioxamine, achromobactin, and petrobactin (Mydy et al., 2020). Siderophore biosynthesis proteins SbnA and SbnB contribute to the iron sparing response of S. aureus that enables staphyloferrin B biosynthesis in the absence of an active tricarboxylic acid cycle (Kobylarz et al., 2014). Region 7th BGC was decorated with the genes belonging to the LuxR family of two-component transcriptional response regulators, RND multidrug efflux transporter, molybdenum ABC transporter permease protein (ModB), and coenzyme PQQ synthesis protein (D, E). The LuxR transcriptional regulator is a key player in quorum-sensing (QS), and coordinates the expression of a variety of genes, including virulence factors, antibiotics biosynthesis, motility, nodulation, plasmid transfer, bioluminescence, and biofilm formation (Chen and Xie, 2011). RND (Resistance-Nodulation-Division) family transporters catalyze the active efflux of many antibiotics and chemotherapeutic agents (Nikaido and Takatsuka, 2009). ModB transporters are involved in the export or import of a wide variety of substrates ranging from small ions to macromolecules. The major function of ABC import systems is to provide essential nutrients to bacteria (Maupin-Furlow et al., 1995).

The 8th of BGC represented the genes belonging to the glutathione S-transferase (GSTs), polyketide synthase (PKSs), acyl-CoA dehydrogenase and synthetases, GMP synthase, and 4′-phosphopantetheinyl transferase. GSTs catalyze the conjugation of the reduced form of glutathione (GSH) to xenobiotic substrates for the purpose of detoxification and consists of three superfamilies; the cytosolic, mitochondrial, and microsomal—also known as MAPEG—proteins (Sheehan et al., 2001; Udomsinprasert et al., 2005). PKSs are involved in the synthesis of polycyclic aromatic natural products having antibacterial, antifungal, anticancerous, antiviral, antiparasitic, and other medicinally significant properties (Wang et al., 2020).

### Gene-related to biotechnological applications

Genomic annotation using the CAZymes database predicted the existence of GHs, GTs, CBMs, and AAs. The annotation also revealed that S-30 possesses genes encoding for cellulase, chitinase, and endoglucanase activity, which play an important role in colonization (Reinhold-Hurek and Hurek, 2011; Mota et al., 2017). Carbohydrate-binding modules facilitate the degradation of complex polysaccharides through polysaccharide hydrolases, whereas AAs can act in concert. Additionally, some genes related to CEs and PLs were identified that can cooperate with GHs to degrade plant polysaccharides (Gavande et al., 2021).

### Analysis of genetic diversity and recombination

Mutational divergence (θ) defined as the mean of mutation numbers per locus since the divergence of a pair of homologous sites is found to have a higher value for *A. pittii* compared to other pathogenic species such as *Y. pestis* (Chen, 2019), *P. aeruginosa* (Lin and Kussell, 2019), *K. pneumoniae* (Lin and Kussell, 2019), *Salmonella enterica* (Park and Cheryl, 2020), and *Staphylococcus pseudintermedius* (Smith et al., 2020). For *A. pittii* stains, recombination divergence (ϕ) and recombination coverage (*c*) had higher values of 9.5 and 0.9, respectively, which is greater than others pathogenic bacterial species (Lin and Kussell, 2019; Park and Cheryl, 2020). In *A. pittii* strain, the relative rate of recombination to mutation (γ/μ) is the only parameter that has shown a lower value of 7.2 compared to γ/μ values in *S. enterica* subsp. *enterica* (Chen, 2019) and *P. aeruginosa* (Park and Cheryl, 2020) estimated to be 9.75 and 11, respectively. In our study, we noted a significant occurrence of high recombination rates and a diverse array of antibiotic resistance genes within *A. pittii* strains. These findings align with previous research that emphasized the substantial role of recombination in the acquisition of antibiotic resistance traits in *Acinetobacter* species (Godeux et al., 2022). The genetic diversity analysis revealed Tajima’s D would be favorable under the assumption that there is a low frequency of rare alleles, which may develop as a result of population bottlenecks or balancing selection (Tajima, 1989; Nei and Kumar, 2000). In addition to that, estimates on Fu’s FS statistics were mostly negative across core genomic regions, which further supports the population expansion hypothesis of *A. pittii* strains (Supplementary Table 5; Chen, 2019).

## Conclusion

In conclusion, the genomic variation of *A. pittii* S-30 in comparison with other *A. pittii* strains has been demonstrated in accordance with the comparative genomics studies. Numerous BGCs clusters of S-30 strains showed both strain-specific and unidentified characteristics, which support the idea that bacteria perform metabolic activity exclusively for survival in a particular ecological environment and potentially construct alternative routes for new bioactive metabolite production. Also, the S-30 gnome carried a set of useful genes that have potential contributions in PGP, the presence of CAZymes, and stress tolerance genes including abiotic and metal stressors, therefore, it can be concluded that S-30 may be used as a potent alternative to chemical fertilizers. Additionally, genome analysis also facilitated the prediction of virulence factors and AMRs in *A. pittii* S-30, which opens the way for future directions to validate the functionality of these candidate genes. Future experimental work is required to validate the functions of these candidate genes and determine their exact pathogenicity using standard cell culture and animal studies.

## Data availability statement

The datasets presented in this study can be found in online repositories. The names of the repository/repositories and accession number(s) can be found in the article/Supplementary material.

## Author contributions

RS: Conceptualization, Data curation, Funding acquisition, Investigation, Methodology, Resources, Software, Supervision, Validation, Writing – original draft. AS: Formal analysis, Investigation, Writing – original draft, Writing – review & editing. SD: Data curation, Formal analysis, Writing – review & editing. KK: Data curation, Formal analysis, Writing – review & editing.

## Footnotes

1
http://www.bioinformatics.babraham.ac.uk/projects/fastqc

2
http://www.cbs.dtu.dk/services/RNAmmer/

3
https://ftp.ncbi.nih.gov/pub/wolf/COGs/

4
http://imedea.uib-csic.es/jspecies/

5
http://www.mgc.ac.cn/VFs/

## Supplementary material

Supplementary Figure 1 **(A)** The growth kinetics study of *A. pittii* S-30 in ZnSO_4_, CuSO_4_, CdCl_2_, HgCl_2_, and NiSO_4_ (each was used at a 5 mM concentration), a control set of tubes without any metal stressors was used as a control. **(B)** Test of motility swimming, swarming and twitching shown by S-30.

Supplementary Figure 2 A core genome-based phylogenetic tree of the *A. pittii* strain was established using a collection of 1,704 single-copy non-recombinant genes, ensuring a robust phylogenetic reconstruction. The genomes in phylogenetic clades that shared similar host types are highlighted with the same color.

Supplementary Figure 3 Pie chart representing the RAST subsystem categories in the *A. pittii* S-30 genome. The most abundant systems on the category level were observed for amino acids and derivatives, followed by protein metabolism, carbohydrate metabolism, and cofactors, vitamins and prosthetic groups.

Supplementary Figure 4 Annotated antibiotic resistance genes in *A. pittii* S-30 genome was identified by CARD (Comprehensive Antibiotic Resistance Database).

Supplementary Figure 5 **(A)** Venn diagram generated by OrthoVenn2 represents the distribution of shared and unique gene clusters among all the six species. **(B)** The bar plot represents the cumulative ortholog clusters found in each species, and it illustrates the cumulative core, shared, and unique clusters in all six species. **(C)** The pairwise protein sequence comparison for the heatmap showing orthologous clusters between OS-1 and other closely related strains.

Supplementary Figure 6 The circular genome comparison of the draft assembly genome of S-30 with the closely related *A. pittii* strains was performed against the reference genome using the BRIG (Blast Ring Image Generator) Tool.

Supplementary Figure 7 IslandViewer was used to determine the genomic island in the S-30 genome. The red represents the prediction by an integrated approach, the blue displays results via IslandPath-DIMOB, and the orange represents genomic islands predicted using SIGI-HMM. The outer black circle represents the scale line in Mbps and the black zigzag line plot delineates each of the contigs identified in strain S-30.
