# Supplementary figures and images for: First report on in-depth genome and comparative genome analysis of a metal-resistant bacterium Acinetobacter pittii S-30, isolated from environmental sample
Source: Front Microbiol. 2024 Apr 29;15:1351161. doi: 10.3389/fmicb.2024.1351161 (PMC11089254; doi:10.3389/fmicb.2024.1351161)

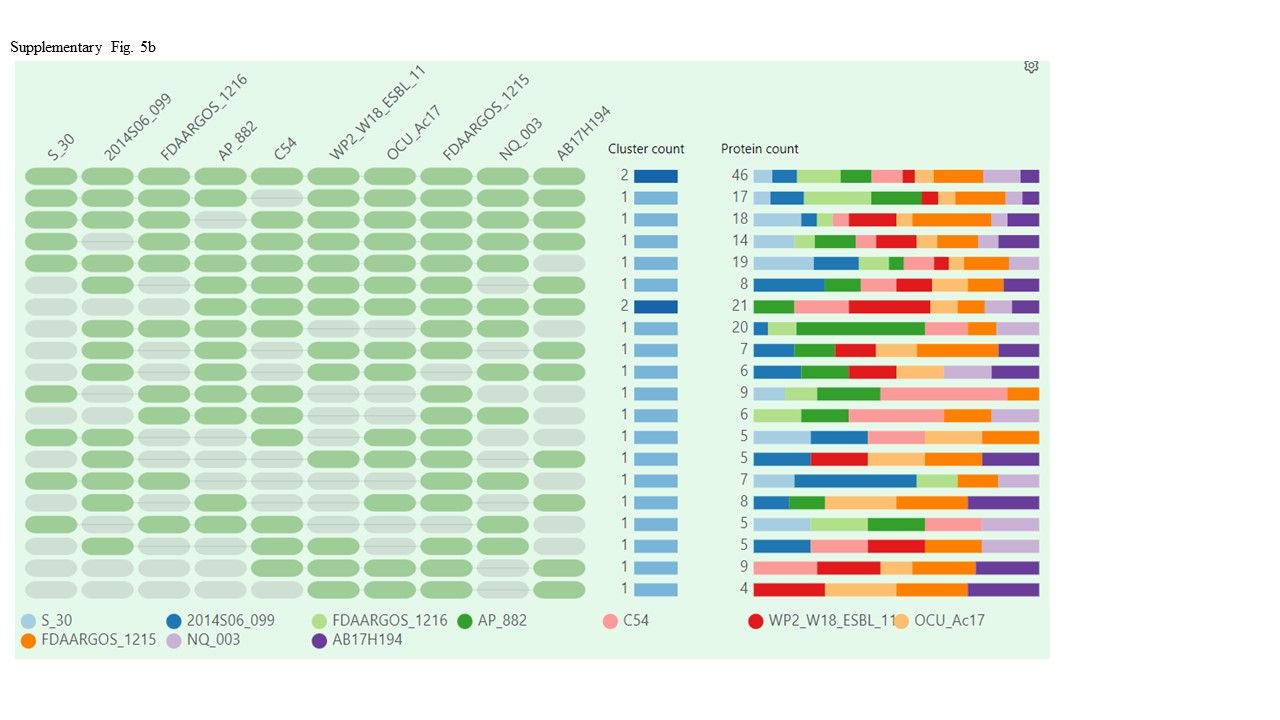

Supplement: Supplementary file 1 [file Image_6.JPEG]

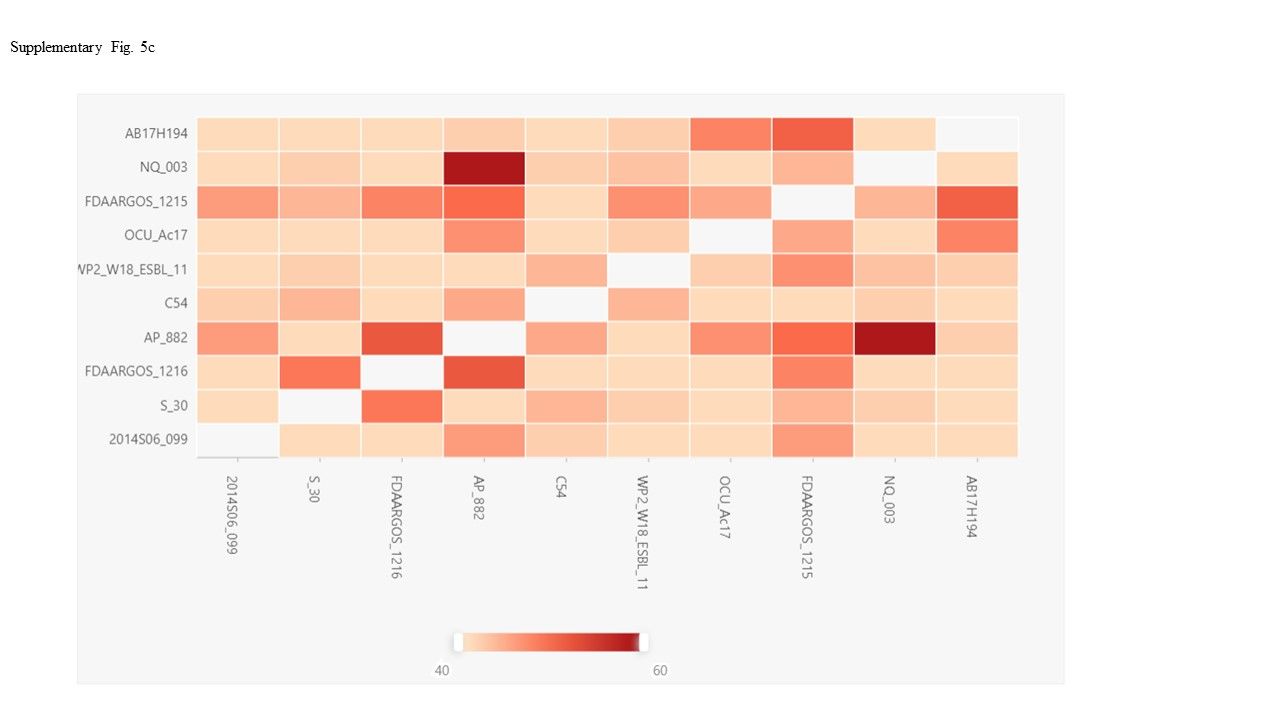

Supplement: Supplementary file 2 [file Image_7.JPEG]

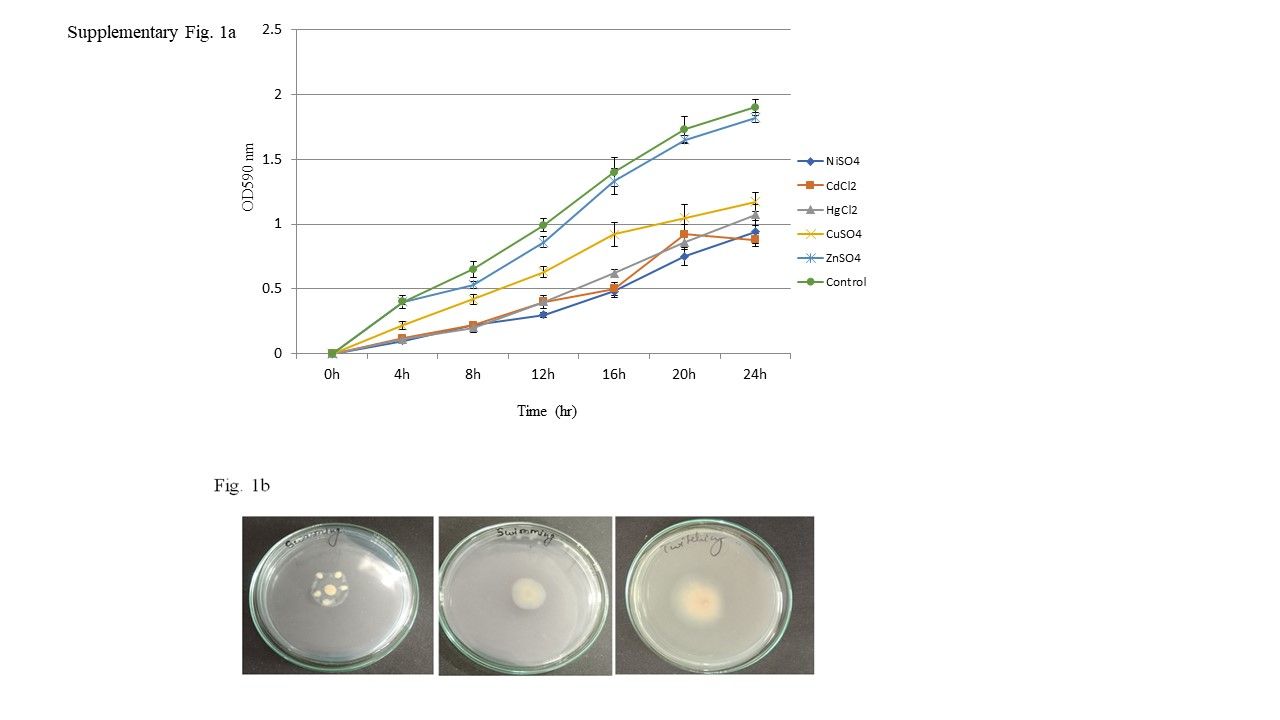

Supplement: Supplementary Figure 1 — (A) The growth kinetics study of A. pittii S-30 in ZnSO4, CuSO4, CdCl2, HgCl2, and NiSO4 (each was used at a 5 mM concentration), a control set of tubes without any metal stressors was used as a control. (B) Test of motility swimming, swarming and twitching shown by S-30. [file Image_1.JPEG]

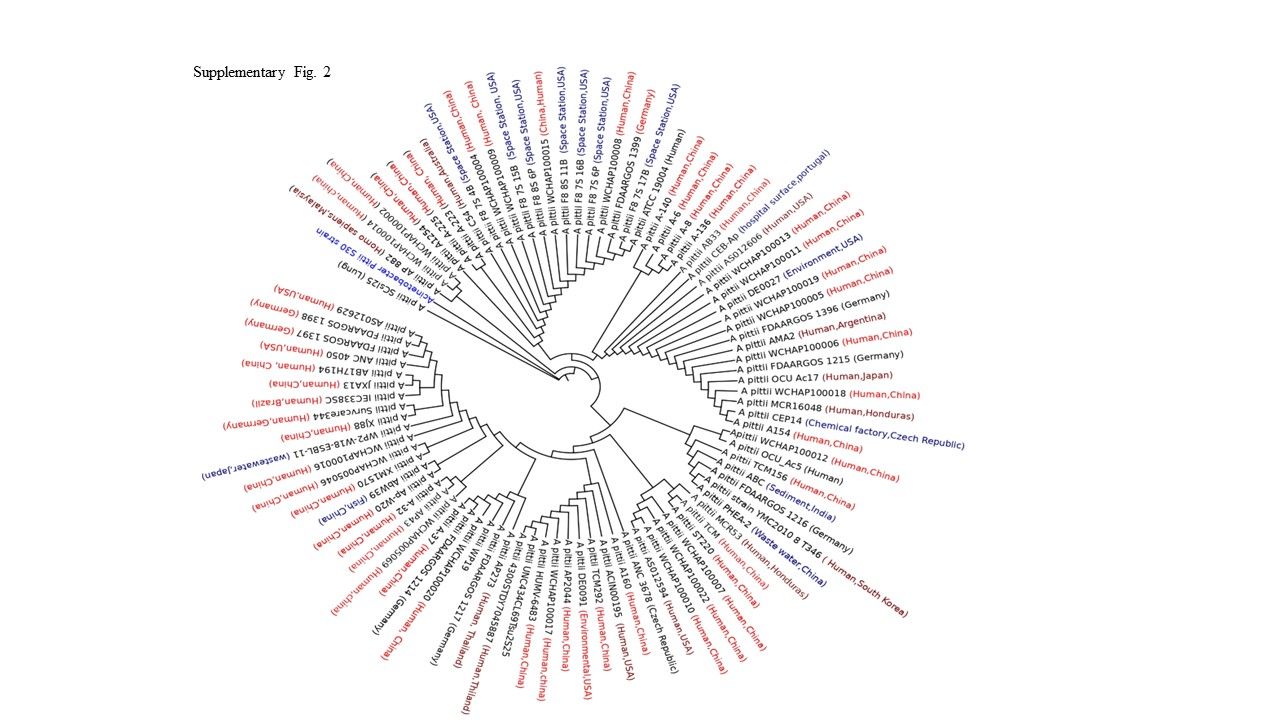

Supplement: Supplementary Figure 2 — A core genome-based phylogenetic tree of the A. pittii strain was established using a collection of 1,704 single-copy non-recombinant genes, ensuring a robust phylogenetic reconstruction. The genomes in phylogenetic clades that shared similar host types are highlighted with the same color. [file Image_2.JPEG]

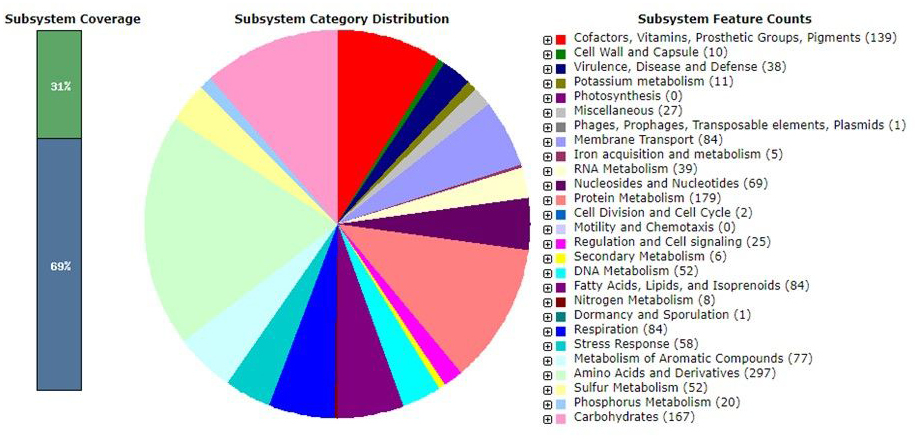

Supplement: Supplementary Figure 3 — Pie chart representing the RAST subsystem categories in the A. pittii S-30 genome. The most abundant systems on the category level were observed for amino acids and derivatives, followed by protein metabolism, carbohydrate metabolism, and cofactors, vitamins and prosthetic groups. [file Image_3.jpg]

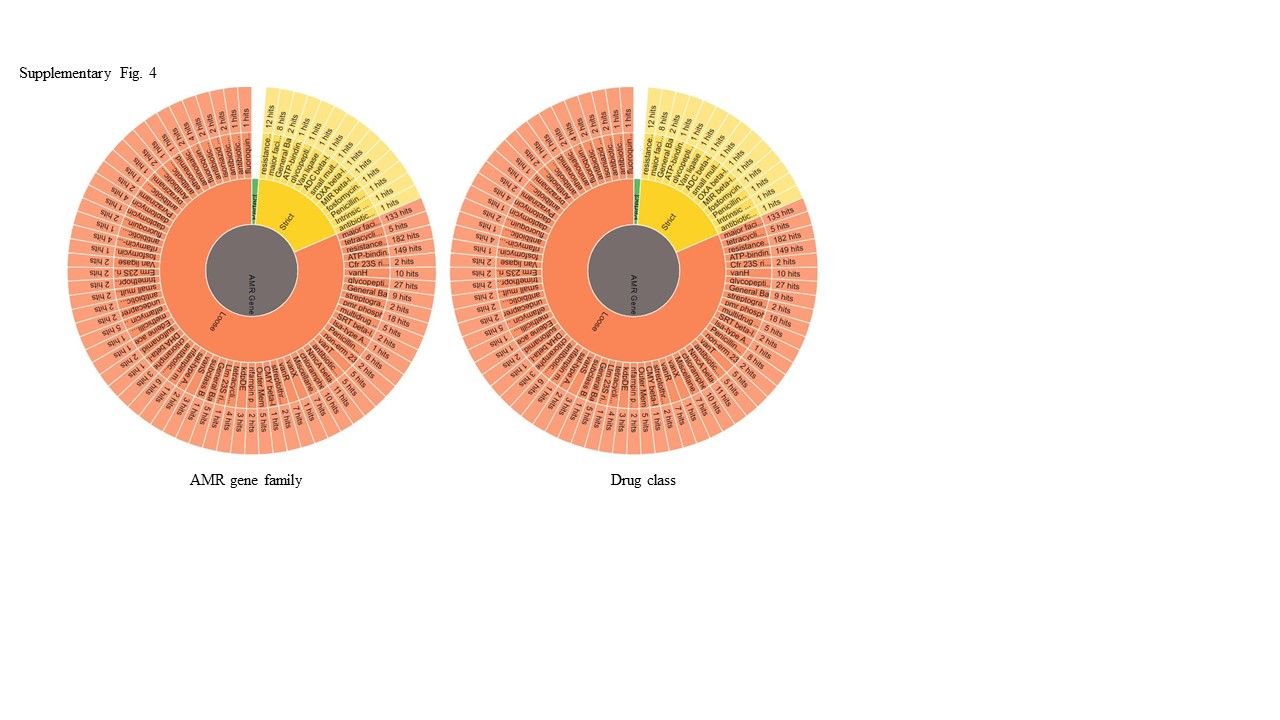

Supplement: Supplementary Figure 4 — Annotated antibiotic resistance genes in A. pittii S-30 genome was identified by CARD (Comprehensive Antibiotic Resistance Database). [file Image_4.JPEG]

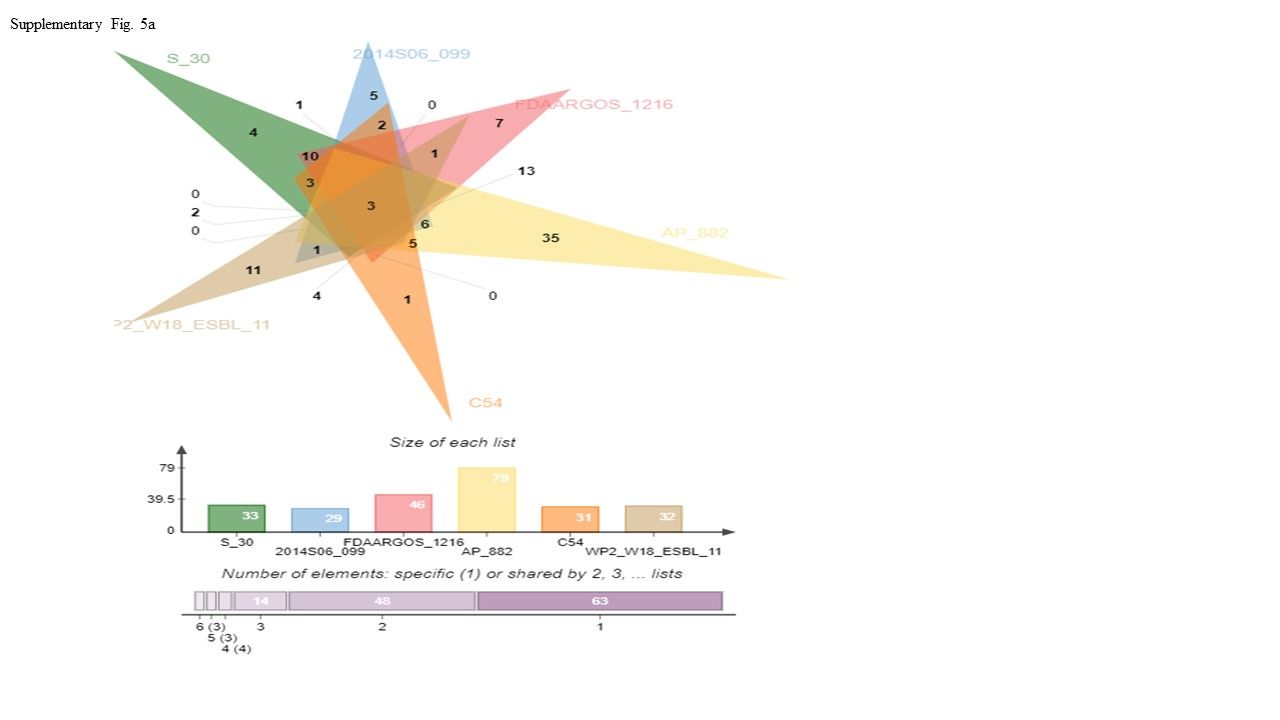

Supplement: Supplementary Figure 5 — (A) Venn diagram generated by OrthoVenn2 represents the distribution of shared and unique gene clusters among all the six species. (B) The bar plot represents the cumulative ortholog clusters found in each species, and it illustrates the cumulative core, shared, and unique clusters in all six species. (C) The pairwise protein sequence comparison for the heatmap showing orthologous clusters between OS-1 and other closely related strains. [file Image_5.JPEG]

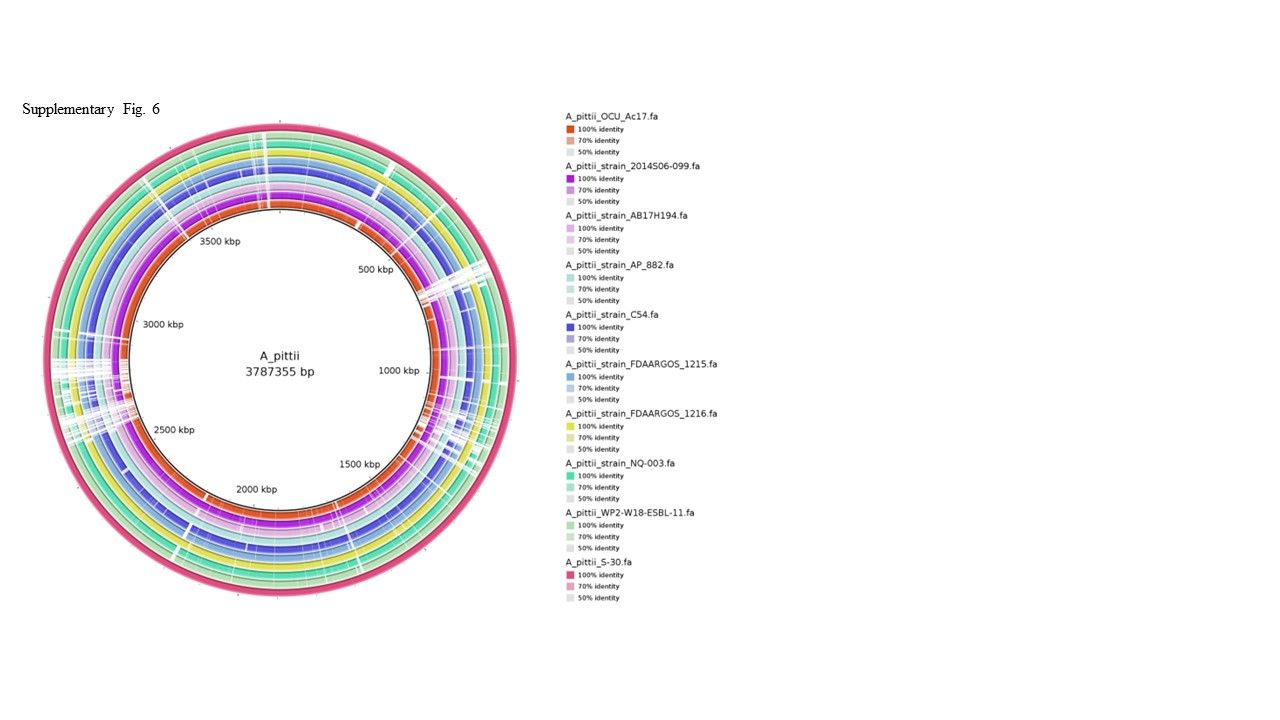

Supplement: Supplementary Figure 6 — The circular genome comparison of the draft assembly genome of S-30 with the closely related A. pittii strains was performed against the reference genome using the BRIG (Blast Ring Image Generator) Tool. [file Image_8.JPEG]

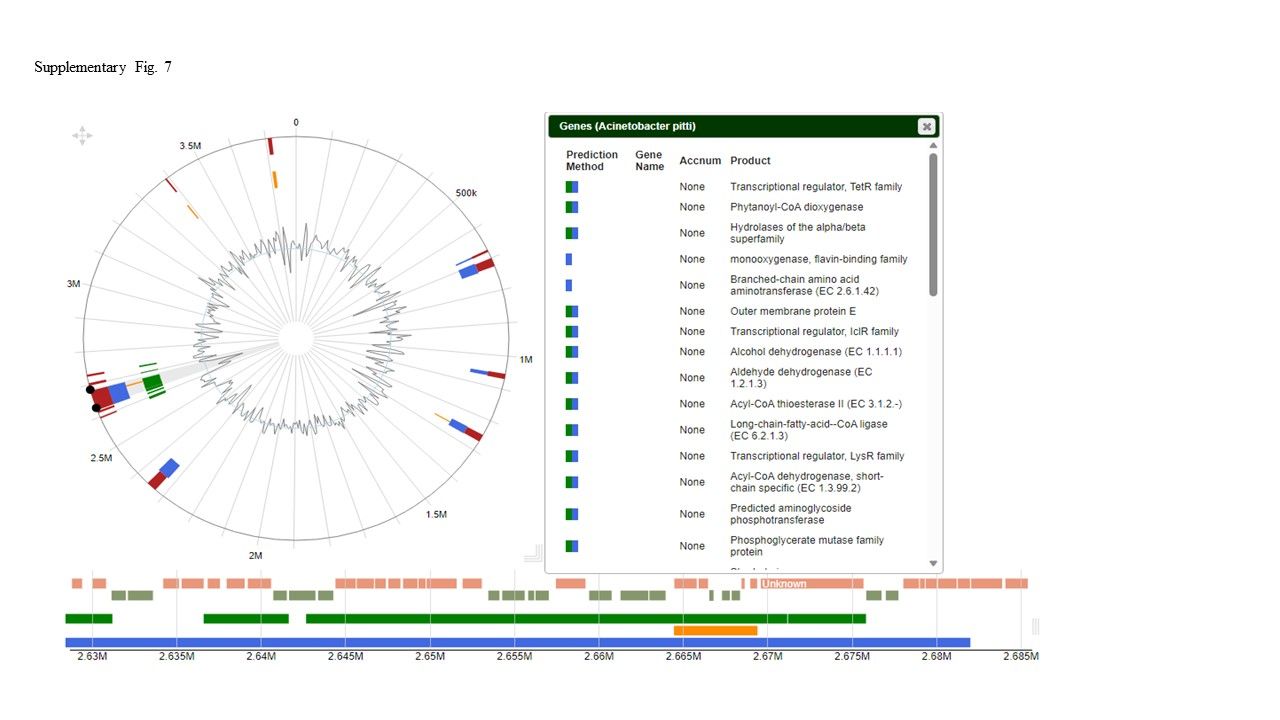

Supplement: Supplementary Figure 7 — IslandViewer was used to determine the genomic island in the S-30 genome. The red represents the prediction by an integrated approach, the blue displays results via IslandPath-DIMOB, and the orange represents genomic islands predicted using SIGI-HMM. The outer black circle represents the scale line in Mbps and the black zigzag line plot delineates each of the contigs identified in strain S-30. [file Image_9.JPEG]
