# Supplementary material for: First report on in-depth genome and comparative genome analysis of a metal-resistant bacterium Acinetobacter pittii S-30, isolated from environmental sample
Source: Front Microbiol. 2024 Apr 29;15:1351161. doi: 10.3389/fmicb.2024.1351161 (PMC11089254; doi:10.3389/fmicb.2024.1351161)
Supplement: Supplementary file 3 [file Table_1.DOCX]

**Supplementary Table 1** Biochemical characteristic feature of *A. pittii* S-30

**Characteristic (s) Activity Carbohydrate Activity**

Gram reaction - Sodium gluconate -

Catalase + Glycerol +

Indole - Salicin -

MR - Dulcitol -

VP - Inositol +

Amylase + Sorbitol -

Lipase + Mannitol -

Pectinase - Adonitol -

Catalase + Inulin +

Cellulase + Arabitol -

Temp. tolerance (°C) 45 Erythritol -

Salt tolerance (%) 6% Citrate +

pH 4-10 α-Methyl-D-glucoside -

Swimming + L-Arabinose +

Swarming + Rhamnose +

Twiching + Cellobiose +

**Carbohydrate Activity** Melezitose +

Lactose + α-Methyl-D-mannoside -

Xylose + Xylitol -

Maltose + ONPG -

Fructose + Esculin hydrolysis +

Dextrose - Mannose +

Sucrose + D-Arabinose -

Galactose + Malonate utilization +

Raffinose _+_ Trehalose +

**Supplementary Table 2** Antagonistic activity of *A. pittii* S-30

**Bacteria Activity Zone of inhibition (mm)**

*Escherichia coli* +++ 19.17±0.33

*Staphylococcus aureus* ++ 13.10±0.12

*Bacillus subtilis* ++ 12.40±0.19

*Salmonella typhi* +++ 17.87±0.20

**Fungal species**

*Aspergillus niger* +++ 17.09±0.29

*Microsporum gypseum* +++ 18.20±0.40

*H. gypsium* ++ 12.20±0.21

*Penicillium citrium* ++ 11.40±0.12

+++ good; ++ moderate, + poor; (±)denote standard deviation; NA no activity

**Supplementary Table 3** Annotated genes for stress responses in S-30 genome

**Gene Functional role**

BetA Choline dehydrogenase

BetB Betaine aldehyde dehydrogenase

BetC Choline-sulfatase

BetT High-affinity choline uptake protein

OpuABC Glycine betaine ABC transport system

CodA Choline oxidase

OpuABCD Choline ABC transport system

GbsB Alcohol dehydrogenase

GbcAB Glycine betaine demethylase

ChAD Choline binding protein

sodA Manganese superoxide dismutase

sodB Superoxide dismutase [Fe]

sodC Superoxide dismutase [Cu-Zn]

NSTR Nitrite-sensitive transcriptional repressor NsrR

GST Glutathione S-transferase

MGS Methylglyoxal synthase

GloA Lactoylglutathione lyase

GloB Hydroxyacylglutathione hydrolase

SAM SAM-dependent methyltransferase

GshA Glutamate--cysteine ligase

GshB Glutathione synthetase

SmoEFGK Various polyols ABC transporter

CysA Sulfate and thiosulfate import ATP-binding protein

FGH S-formylglutathione hydrolase

FrmR Transcriptional regulator of formaldehyde detoxification operon

**Supplementary Table 4** Annotated genes for phosphate metabolism in S-30 genome

**Gene Functional role**

pstS Phosphate ABC transporter

PstA Phosphate transport system permease protein PstA

PstB Phosphate transport ATP-binding protein

PhoP Alkaline phosphatase synthesis transcriptional regulatory protein

PhoR Phosphate regulon sensor protein

PhoB Phosphate regulon transcriptional regulatory protein

phoQ response regulator in two-component regulatory system

PhoU Phosphate transport system regulatory protein

PhnRSTUV 2-aminoethylphosphonate ABC transporter permease protein

PhnW 2-aminoethylphosphonate:pyruvate aminotransferase

PhnX Phosphonoacetaldehyde hydrolase

PhnA Phosphonoacetate hydrolase

PtxD Phosphonate dehydrogenase

PtxE LysR-family transcriptional regulator

**Supplementary Table 5** Annotated genes for sulfur metabolism in S-30 genome

**Gene Functional role**

CysTW Sulfate transport system permease protein

CysP Sulfate and thiosulfate binding protein

SulP Sulfate permease

CysZ Sulfate transporter

SulP2 Sulfate permease

SAT12 Sulfate adenylyltransferase

PAPSR Phosphoadenylyl-sulfate reductase

ASK Adenylylsulfate kinase

APSR Adenylyl-sulfate reductase

ASR_AB Adenylylsulfate reductase

TrxR Thioredoxin reductase

AhpCF Alkyl hydroperoxide reductase protein

AhpD Alkylhydroperoxidase protein

SsuA Alkanesulfonates-binding protein

SsuD Alkanesulfonate monooxygenase

SsuB Alkanesulfonates ABC transporter ATP-binding protein

SsuF Organosulfonate utilization protein

DDEB Dibenzothiophene desulfurization enzyme

ASR Putative arylsulfatase regulatory protein

AstR Sulfate ester binding protein

AtsK Putative alkylsulfatase

**Supplementary Table 6** Annotated genes for metabolism of aromatic compounds in S-30 genome

**Gene Functional role**

dmpLMNOP Phenol hydroxylase

QuiA Quinate/shikimate dehydrogenase

QuiBC 3-dehydroquinate dehydratase

BenABCD Benzoate 1,2-dioxygenase beta subunit

BenK benzoate MFS transporter BenK

BenE2 Benzoate transport protein

BenF benzoate-specific porin

BenR benABC operon transcriptional activator

CatR Aromatic hydrocarbon utilization transcriptional regulator

OhbA Ortho-halobenzoate 1,2-dioxygenase beta-ISP protein

ClBD 2-chlorobenzoate 1,2-dioxygenase

pobA P-hydroxybenzoate hydroxylase

CatA Catechol 1,2-dioxygenase

CatB Muconate cycloisomerase

CatC Muconolactone isomerase

CatD Beta-ketoadipate enol-lactone hydrolase

CatEA 3-oxoadipate CoA-transferase

SalA Salicylate hydroxylase

SalE Salicylate esterase

AreB AreB (Aryl-alcohol dehydrogenase)

AreC Benzaldehyde dehydrogenase

maoA Monoamine oxidase

maoC Aldehyde dehydrogenase
